# Prevalence and trends of contact sensitization in patients with psoriasis in Lithuania

**DOI:** 10.1016/j.jdin.2024.02.015

**Published:** 2024-04-06

**Authors:** Gabija Rudzikaitė-Fergizė, Augustė Senulytė, Neringa Guobytė, Andrius Jurėnas, Monika Macejevska, Jūratė Grigaitienė

**Affiliations:** a Clinic of Infectious Diseases and Dermatovenereology, Vilnius University Faculty of Medicine, Vilnius, Lithuania; b Centre of Dermatovenereology, Vilnius University Hospital Santaros Klinikos, Vilnius, Lithuania; c Vilnius City Clinical Hospital, Vilnius, Lithuania

**Keywords:** allergen, contact sensitization, cutaneous allergy, European Baseline Series, prevalence, psoriasis

## Abstract

**Background:**

Psoriasis and allergic contact dermatitis are 2 very common dermatoses. The relationship between them has not yet been fully understood. Contact dermatitis can be an additional cause of epidermal disruption in psoriasis patients, resulting in poor management of the disease.

**Objective:**

To analyze the tendencies of contact sensitization in a cohort of psoriasis patients with suspected allergic contact dermatitis.

**Methods:**

Psoriasis patients (*n* = 85) with suspected contact dermatitis underwent patch testing with European Baseline allergen series S-1000 in Vilnius University Hospital Santaros Klinikos Centre of Dermatovenereology from August 2020 to August 2021. Their results are presented in this study.

**Results:**

The patch test was positive in 43.5% (*n* = 37) of patients. Contact sensitization was more prevalent in patients with mild psoriasis, as characterized by Psoriasis Area Surface Index scores ≤10, compared to those with moderate-to-severe psoriasis (*P* < .05). Generalized psoriasis and nail involvement were more common among nonsensitized patients (*P* < .05). Most common contact allergens among sensitized patients were nickel (II) sulfate, formaldehyde, and potassium dichromate.

**Conclusion:**

An inverse trend was observed between psoriasis severity and contact sensitization. Extended psoriatic involvement was uncommon in sensitized patients.

**Capsule Summary**

• Contact sensitization to allergens was more prevalent in patients with Psoriasis Area Surface Index score ≤10.
• Most common allergens among sensitized patients were nickel sulfate, formaldehyde, and potassium dichromate.

## Introduction

Allergic contact dermatitis (ACD) and psoriasis are highly prevalent inflammatory skin disorders characterized by epithelial alterations and deviated T cell immunity.^1^ Main characteristics of psoriasis are squamous, well-demarcated erythematous plaques of variable size, which are the result of abnormal proliferation and differentiation of keratinocytes and a dysregulation of immune cell activation in the dermis and epidermis.^2^ Meanwhile, ACD is mainly distinguished by erythema, papules, and vesicles followed by scaling, resulting from type IV hypersensitivity induced keratinocyte apoptosis.^1^^,^^3^ Despite the distinct pathophysiology of these 2 conditions, they can cooccur, with contact allergy being a coexisting condition in psoriasis patients in about 20% to 25% of cases.^4^ Contact dermatitis can result in poorer management of psoriasis due to additional epidermal disruption. However, the data on the association between these 2 conditions remains controversial.^5^ This study aims to evaluate the prevalence of contact sensitization among a cohort of psoriasis patients with suspected ACD and to investigate potential risk factors associated with this condition.

## Material and methods

This cross-sectional study includes the data of 85 patients with psoriasis and suspected contact dermatitis collected in the Vilnius University Hospital Santaros Klinikos Centre of Dermatovenereology from August 2020 to August 2021. Study participants filled out an original questionnaire, which included questions about the personal history of psoriasis, contact dermatitis and chronic health conditions, personal and family history of allergy and atopy, flare factors for psoriasis, topical skincare, medications, and occupational/hobby exposures. Patients underwent a detailed examination by a dermatovenereologist, who confirmed the diagnosis of psoriasis according to clinical and dermoscopic findings and evaluated the extent of involvement. Psoriasis Area Surface Index (PASI) and Dermatology Life Quality Index scores were calculated for each patient. Severity of psoriasis characterized by PASI was considered mild when PASI was ≤10, and moderate-to-severe—PASI >10. Skin patch testing was performed with European baseline allergen series S-1000 obtained from Chemotechnique Diagnostics (Vellinge) and Finn Chambers chambers (8 mm; Epitest), which were applied using Scanpor (Norgesplaster) tape and taken off after 48 hours. The readings were performed on days 3, 4, and 7 and outcomes were concluded according to the guidelines of the European Society of Contact Dermatitis.^6^ Allergens were categorized into these categories: metals (1,5,7), preservatives (10,18,21,23,26,29), fragrances (15,19,27,28), dyes (2,30), pharmaceuticals (4,6,24,25), rubber additives (3,11,13,17), resins (9,14,16), and other (12,20,22). Laboratory and joint imaging test results were sourced from medical records.

The study protocol was approved by the Vilnius Regional Biomedical Research Ethics Committee (No. 2020/8-1242˗729). Written consent was taken from each patient who participated in the study.

Data analysis was performed using Microsoft Excel version 16.67 and IBM SPSS version 29.0.0.0 software (IBM). Qualitative variables were presented using absolute frequency (*n*) and percentage (%) of the analyzed sample. Pearson’s χ^2^ test and Fisher’s exact criterion were used to compare the frequencies of qualitative variables. The normality of the distribution of continuous variables was tested using Kolmogorov-Smirnov test and Shapiro-Wilk test for the sensitized patient sample (<50 patients).^7^ The nonparametric Mann-Whitney U test was used to compare quantitative variables between groups. Results were considered statistically significant if *P* was less than .05. Microsoft Excel and Microsoft Word software was used to provide visual and graphical data.

## Results

### Characteristics of the cohort

Out of 85 patients, 54 (63.5%) were female and 31 (36.5%) were male, with a mean age of 50.68 ± 15.298, body mass index—28.69 ± 6.53. Mean PASI and Dermatology Life Quality Index scores were 9.50 ± 6.17 and 8.86 ± 6.34 respectively. The general characteristics of psoriatic lesions are presented in Table I. In 43.5% (*n* = 37) of cases the patch test was positive. The maximum sensitivity to 8 allergens was detected in 2 patients. The prevalence of skin sensitization was higher in women than in men (75.7% vs 24.3%, *P* = .041). At the time of the study, a total of 3 patients were being treated with biological therapy and 14 patients were receiving methotrexate.

**Table I:** General characteristics of psoriatic lesions

	*n*	%
Psoriasis severity based on PASI score		
Mild psoriasis	47	55.3
Moderate-to-severe psoriasis	38	44.7
Localization of psoriasis lesions		
Scalp	52	61.2
Face	31	36.5
Trunk	49	57.7
Arms	58	68.2
Legs	59	69.4
Palms	55	64.7
Soles	51	60.0
Extented involvement		
Nail involvement	63	74.1
Joint involvement	39	45.9

*PASI*, Psoriasis Area Surface Index.

### Differences between positive and negative patch test groups

**Fig 1:**
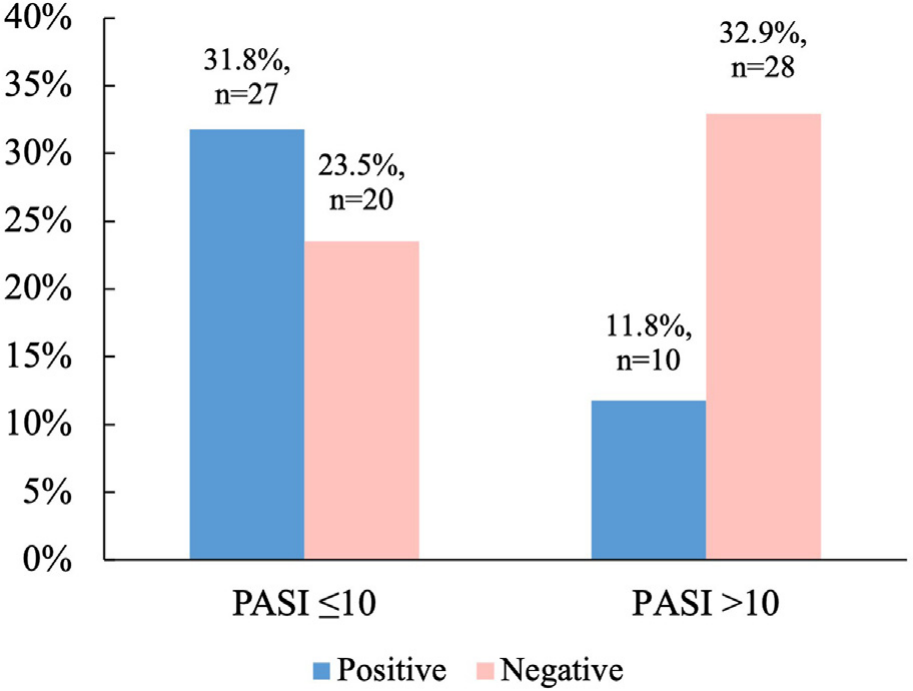
Patch testing results in psoriasis severity groups characterized by PASI score. Mild psoriasis was considered if PASI score was ≤10. Moderate-severe psoriasis was considered if PASI score >10. *PASI*, Psoriasis Area Surface Index.

**Fig 2:**
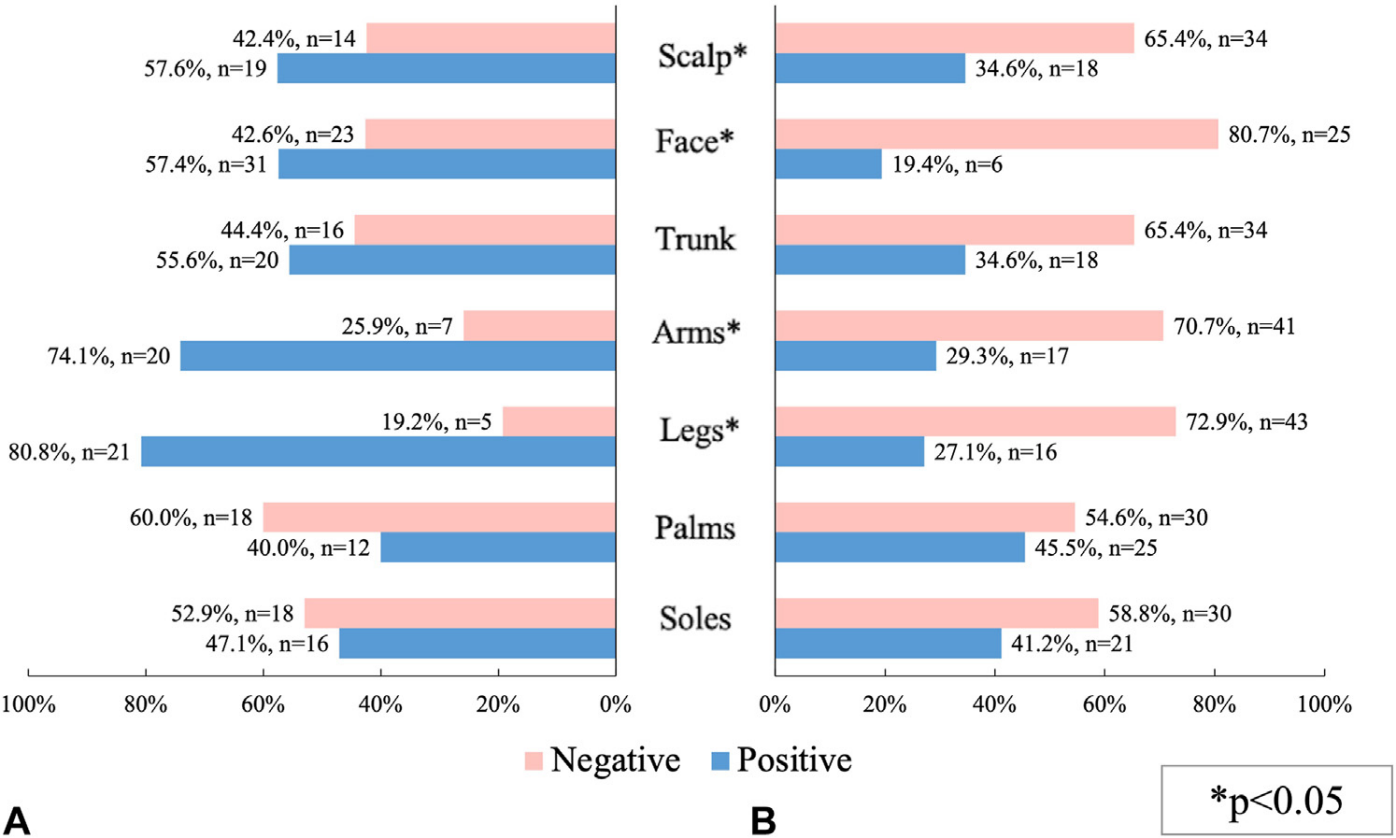
**A,** Negative and positive test ratio *without* psoriatic lesions in specified regions. **B,** Negative and positive test ratio *with* psoriatic lesions in specified regions.

Severity of psoriasis characterized by PASI score was associated with patch testing results (*P* = .004) (Fig 1). There was also an association between a shorter personal history of psoriasis and positive patch test result (*P* = .001). Sensitized patients had significantly lower PASI scores than those with negative skin patch tests (mean rank 49.1 vs 35.1, U = 596.00, *P* = .01), while there was no difference in Dermatology Life Quality Index scores between the 2 groups. Skin sensitization was less prevalent in patients with psoriatic plaques on their arms, legs, scalp, face (*P* < .001, *P* < .001, *P* = .034, and *P* < .001 respectively) (Fig 2). Nail psoriasis, especially pitting of the nails and toe involvement were more prevalent in those who had negative patch test results (*P* = .007, *P* = .053, and *P* = .009 respectively). Patients without contact sensitization on average had a higher number of affected nails than patients who were sensitized (mean rank 47.5 vs 36.6, U = 649.500, *P* = .033). Joint involvement did not differ significantly between the 2 groups.

Family history of bronchial asthma was associated with a higher proportion of positive patch test results (*P* = .051). No statistically significant difference was observed between other diseases, laboratory test results, hobby/occupational exposures and patch test results.

### Tendencies in sensitized patients

**Fig 3:**
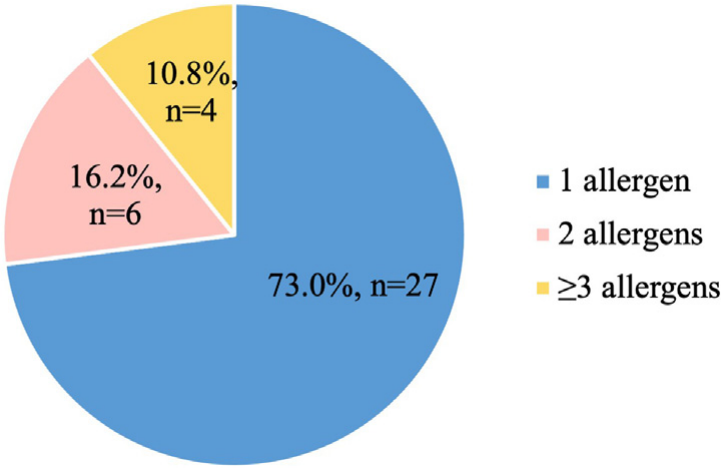
Number of allergens per patient.

**Fig 4:**
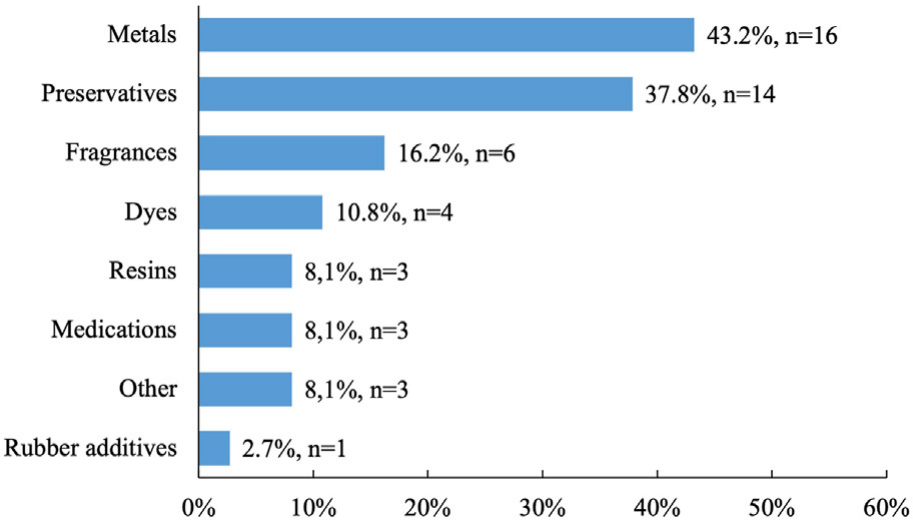
Most common contact allergen groups.

**Fig 5:**
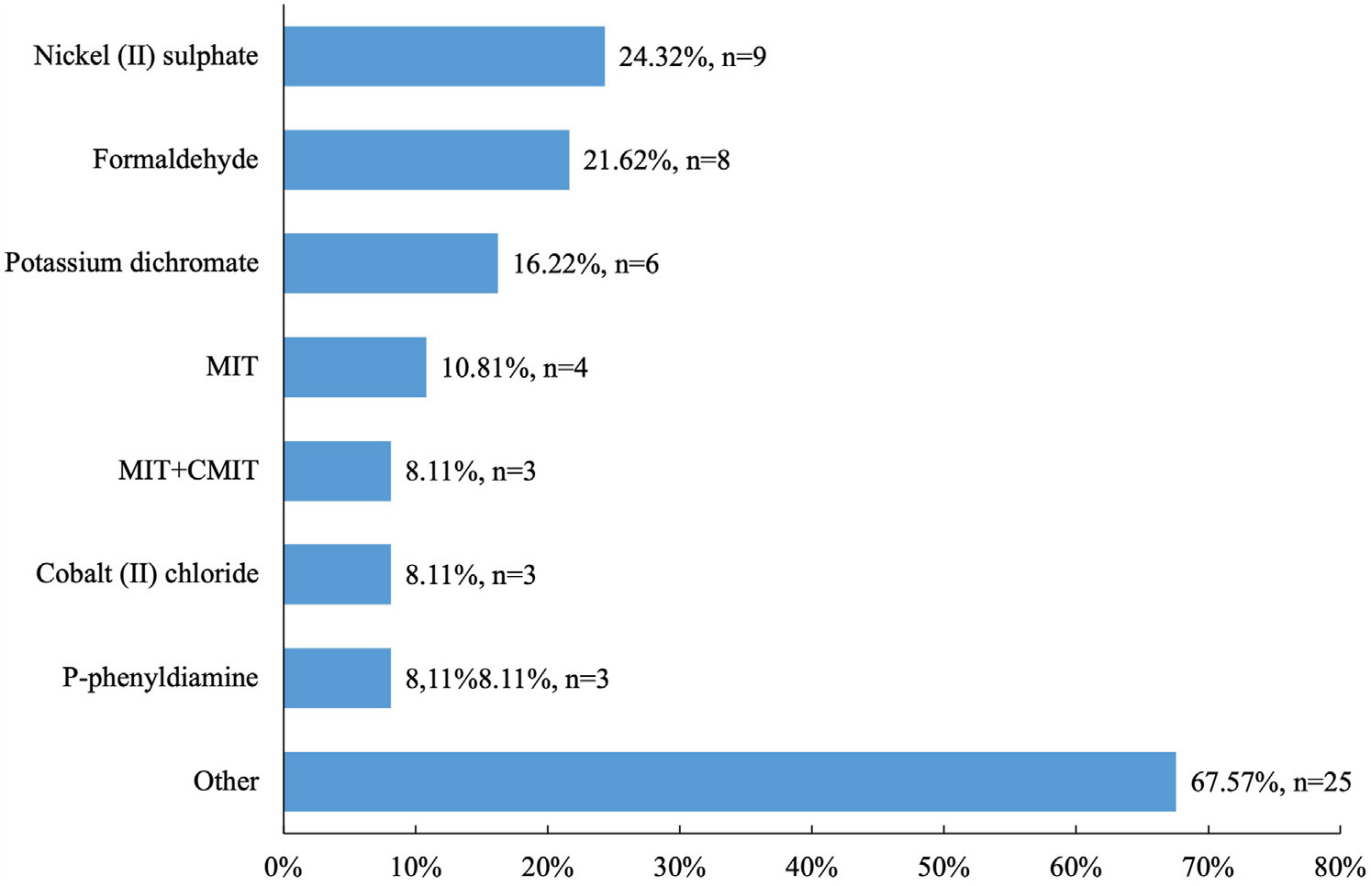
Most common contact allergens. *CMIT*, Methylchloroisothiazolinone; *MIT*, methylisothiazolinone.

The majority (73.0%, *n* = 27) of patients had sensitization to 1 allergen (Fig 3). In our cohort, contact allergy to metals was the most prevalent, with 16 patients (43.2%) having sensitivity to at least 1 allergen that belongs to this group (Fig 4). The most common allergens were nickel (II) sulfate (24.3%, *n* = 9), formaldehyde (21.6%, *n* = 8), and potassium dichromate (16.2%, *n* = 6) (Fig 5). Contact allergy caused by compounds frequently used in cosmetics (2,9,10,12,15,18,19,20,21,23,26,27,28,29) was less common in those with persistent psoriasis lesions (*P* = .009).

## Discussion

Our study found that approximately 50% of the patients had a positive reaction to at least 1 allergen. The positive test ratio was significantly higher in patients with mild psoriasis, as determined by PASI score ≤10, compared to those with moderate-to-severe psoriasis. Contact sensitization was less prevalent in patients with generalized psoriasis or nail involvement and in those with longer personal history of the disease, suggesting an inverse association between psoriasis severity, length of the disease and allergen sensitization.

Controversial data exists regarding the relationship between psoriasis and ACD. On one hand, the coexistence of contact sensitization with psoriasis may explain the localization of some lesions, treatment resistance and prognosis with increased skin disease morbidity.^1^ Jo et al reported a case of Koebner phenomenon due to allergic contact hypersensitivity and suggested investigating for possible contact sensitization if the pruritic lesions involve pustules and vesicles.^8^ However, several epidemiological and cohort studies have reported an inverse relationship between the 2 diseases.^5^^,^^9^, ^10^, ^11^, ^12^, ^13^ On immunological level, psoriasis development is driven by the excessive activation of the adaptive immune system. A variety of cell types, including plasmocytoid dendritic cells, keratinocytes, natural killer T cells, and macrophages, secrete cytokines such as tumor necrosis factor α, which activate myeloid dendritic cells. These cells then start secreting interleukin (IL)-12 and IL-23, which play an important role of T helpers (T_h_) 1, T_h_17, and T_h_22 cell activation. IL-23 pathway is mediated intracellularly via Tyrosine kinase 2-Janus kinase 2 and signal transducer and activator of transcription 3, which leads to transcription of key inflammatory factors. T_h_1, T_h_17, and T_h_22 cells secrete interferon γ, tumor necrosis factor α, IL-17 and IL-22, which are the cytokines responsible for the keratinocyte proliferation, increased expression of angiogenic mediators, endothelial adhesion molecules, and lesional infiltration of immune cells.^14^ Histological assessments of psoriasis samples reveal epidermal hyperplasia with elongated rete ridges, parakeratosis associated with focal orthokeratosis, and the presence of neutrophil (Munro) abscesses, as well as a disappearance of the granular layer of the epidermis and T-cell infiltrate.^1^ These findings align with the results of in vivo and in vitro experiments, which show that keratinocytes in psoriasis patients are less susceptible to apoptosis.^15^^,^^16^ On the other hand, ACD is a type IVa hypersensitivity reaction, characterized by 2 distinct phases. The sensitization phase occurs when the sensitizing chemical binds to cellular proteins and forms a hapten-protein complex, activating antigen-presenting cells. These hapten-bearing antigen-presenting cells then travel to regional lymph nodes where they prime naive T lymphocytes, leading to the differentiation of memory or effector T cells. The elicitation stage takes place after reexposure of a sensitized individual to the same haptens, which are taken up by keratinocytes and presented to effector or memory T cells. As a response to the contact with the presented hapten, infiltrating T cells release interferon γ, IL-4, tumor necrosis factor α, and IL-17. The latter is one of the key effectors in psoriatic lesion formation. Moreover, a significantly higher levels of IL-17 positive T cells were observed in psoriatic plaques compared to ACD lesions.^3^ In response to interferon γ, keratinocytes upregulate adhesion molecules and chemokines, which further increases the recruitment of immune cells to the area. An inflammatory response is mounted to eliminate antigen-modified keratinocytes, which undergo apoptosis, resulting in loss of cell cohesion, tissue destruction, and desquamation.^17^ In pathology sections, lesions associated with ACD display T cell infiltration in the upper dermis and spongiosis primarily in the lower epidermis, with keratinocyte apoptosis. The hallmark of ACD, apoptotic processes, are in contrast to the apoptosis-resistant and metabolic-active epidermis that is characteristic of psoriasis.^1^ Additionally, the migration of antigen-presenting cells into the local lymph node is delayed in patients with psoriasis.^18^ A previous study investigating the characteristics of ACD in psoriasis patients reported delayed patch test reactions compared to nonpsoriatic individuals, with the average peak reaction intensity occurring after 7 days in psoriasis patients compared to 3 to 5 days in the control group.^3^ For this reason, an additional reading of patch tests was added on day 7 and patients were encouraged to contact the research team if they noticed any new reactions after the final reading. The opposing mechanisms of these 2 conditions may explain the inverse relationship between the severity of psoriasis and contact sensitization to allergens. Sorenson et al reported a case of ACD caused by urushiol, with sparing of exposed psoriatic plaques. The authors argue that the hyperkeratosis and immunological environment of the active psoriasis plaque helped to prevent the patient from developing a typical hypersensitivity reaction in areas affected by psoriasis.^19^

There are only a very limited number of similar studies that have investigated the relationship between psoriasis severity and characteristics and contact sensitization. Palmoplantar psoriasis is suspected to coexist with and be aggravated by contact allergy.^1^^,^^20^ Also, face is a common location for contact allergy, especially in women.^21^ In our study there was no association between palmoplantar lesions and sensitization to allergens, while other regions of the body (face, scalp, trunk, arms, and legs) were less likely to be affected by psoriasis in sensitized patients compared to those with negative patch test results. Regarding the cases when palmoplantar lesions are present, the differential diagnosis between nonpustular psoriasis, ACD, and a combination of these 2 conditions can be difficult.^22^

Regarding the tendencies among sensitized patients, the most common allergen group was metals. The most frequent allergens were nickel (II) sulfate, formaldehyde, and potassium dichromate. The results are similar to another single-center study conducted in Lithuania, which analyzed the data regarding contact sensitization in 1425 patients with suspected contact allergy. It reported that among the allergens in the European Baseline series S-100 2019, metals and preservatives were the most common allergen groups, and nickel (II) sulfate, methylisothiazolinone, and formaldehyde were the most frequent allergens identified in the study sample.^23^ Other studies also report nickel (II) sulfate as one of the most common culprits causing contact sensitization in psoriasis patients. Other frequently detected allergens reported in these studies include methylisothiazolinone, fragrance mix-1, cobalt (II) chloride, potassium dichromate.^5^^,^^24^, ^25^, ^26^, ^27^

The present study has several limitations that must be acknowledged. Firstly, the cross-sectional design of the study does not provide evidence for a causal relationship between the factors explored and contact sensitization. Further research is necessary to establish causality between these variables. Moreover, some patients that were pan-negative to the patch test, could also have contact sensitization to allergens which were not present in the series. While some studies have shown that treatment with methotrexate is not likely to interfere with patch testing results, due to the fact that its effect is dose-dependent, it could have still made an influence on our results.^28^^,^^29^ Furthermore, while other potential causes of psoriasiform lesions in the differential diagnosis could not be ruled out, it is crucial to note that patients included in this study exhibited a typical presentation of psoriatic lesions. Also, a larger cohort would enhance the accuracy of statistical analysis.

## Conclusion

In conclusion, an inverse relationship was observed between psoriasis severity and the extent of psoriatic involvement. Results indicated that contact sensitization was more prevalent in patients with mild psoriasis, as characterized by lower PASI scores, compared to those with moderate-to-severe psoriasis. Most common contact allergens were nickel (II) sulfate, formaldehyde, and potassium dichromate. Further research is needed to better understand the relationship between psoriasis severity and ACD, and the associated risk factors.

## Conflicts of interest

None disclosed.
